# Epidemiology of latent tuberculosis infection in Japan-born and foreign-born children in Japan

**DOI:** 10.5365/wpsar.2023.14.4.1008

**Published:** 2023-11-23

**Authors:** Saori Kasuya, Akiko Imai, Kazuhiro Uchimura, Akihiro Ohkado, Lisa Kawatsu

**Affiliations:** a Research Institute of Tuberculosis, Japan Anti-Tuberculosis Association, Tokyo, Japan.; b Nagoya City University, Aichi, Japan.

## Abstract

**Objective:**

This study aims to compare the epidemiology of notifications of latent tuberculosis infection (LTBI) among Japan-born and foreign-born children in Japan between 2010 and 2020, and to assess the language used during LTBI case interviews with parents or caregivers of foreign-born children with LTBI during 2019.

**Methods:**

Our study consisted of two parts: (1) an analysis of national data from the Japan Tuberculosis Surveillance (JTBS) system on the epidemiology of LTBI among Japan-born and foreign-born children in Japan, and (2) a survey of staff at public health centres that had registered at least one foreign-born child aged ≤ 14 years with LTBI. Data were extracted from the JTBS system for all children aged ≤ 14 years who were newly notified as having LTBI between 2010 and 2020, and analysed to determine trends, characteristics and treatment outcomes. Staff at relevant public health centres completed a self-administered survey.

**Results:**

A total of 7160 Japan-born and 320 foreign-born children were notified as having LTBI between 2010 and 2020. Compared with Japan-born children, foreign-born children notified as having LTBI were more likely to be older, have their mother or sibling as their source of infection and have LTBI detected via a routine school health check. At case interviews, the use of language interpretation services was limited, even when both parents were non-Japanese. No interview was directly conducted with children themselves, not even with school-aged children.

**Discussion:**

Foreign-born children and their parents may be unfamiliar with the system of testing for TB infection and the diagnosis of LTBI in Japan in school settings. Public health centres are required to provide education to patients and their families and care that takes into account cultural and linguistic differences. However, the provision of language support during case interviews may need strengthening.

Japan has a low burden of tuberculosis (TB), with 11 519 cases newly notified in 2021, for a rate of 9.2/100 000 population. (*1*) Although both the number of and the notification rate for TB cases have been steadily declining, the burden of TB among foreign-born persons has been increasing. (*1*) In 2021, the proportion of foreign-born persons among total TB cases was 11.8%; however, this proportion was 68.4% among those aged 15–24 years and 67.1% among those aged 25–34 years. Approximately 80% of cases of TB among foreign-born people in Japan occur in people from six Asian countries: China, Indonesia, Myanmar, Nepal, the Philippines and Viet Nam. Slightly more than one third are notified as having TB within 2 years of entering Japan. (*1*)

Latent tuberculosis infection (LTBI) is also notifiable in Japan, and as with active TB, once notified, its treatment is publicly funded and patients receive adherence support from public health centres (PHCs), which are responsible for registering and managing treatment support for persons diagnosed with TB and LTBI. The epidemiology of LTBI follows that of active TB, whereby the proportion of foreign-born persons notified with LTBI has continued to increase. (*2*) As most cases of LTBI among foreign-born persons are diagnosed among those aged 15–34 years, more attention has been paid to providing care and treatment for adults. (*3*, *4*) However, a consistent number of LTBI cases have been diagnosed among foreign-born children in Japan. Patient-centred care and treatment for children with LTBI involve not only children themselves but also their parents or caregivers. However, little is known about the treatment or support provided to foreign-born children with LTBI in Japan.

The objectives of our study were to compare the epidemiology of LTBI notifications among foreign-born and Japan-born children in Japan between 2010 and 2020, and to assess the language used during LTBI case interviews with parents or caregivers of foreign-born children with LTBI during 2019.

## Methods

Our study consisted of two parts: (1) an analysis of national data from the Japan Tuberculosis Surveillance (JTBS) system about the epidemiology of LTBI among Japan-born and foreign-born children in Japan, and (2) a survey of staff at PHCs in Japan that had registered at least one foreign-born child aged ≤ 14 years with LTBI.

### Analysis of notification data

LTBI has been notifiable in Japan since 2007. In 2017, the JTBS system underwent several major revisions, one of which enabled cohort analysis for all types of TB and LTBI, which was previously possible only for pulmonary TB.

Data were extracted from the JTBS system for all children aged ≤ 14 years who were newly notified with LTBI between 2010 and 2020. Treatment outcomes were extracted for those notified between 2016 and 2019. The period 2016–2019 was chosen for cohort analysis since treatment outcomes for LTBI became available only from 2016. Trends and characteristics were summarized descriptively using numbers and proportions. Treatment outcomes included “treatment success,” “died,” “treatment failed,” “lost to follow up,” “transferred out,” “still in treatment” and “unknown.” Appropriate variables were compared between foreign-born children and Japan-born children using the χ^2^ test with Bonferroni corrections.

### Survey of public health centres

All PHCs that had registered at least one foreign-born child aged ≤ 14 years with LTBI during 2019 were identified from the JTBS system. A self-administered survey was sent by e-mail to TB personnel at each of these PHCs. The survey consisted of questions about the basic demographics of the child (or children), parents or caregivers, and the language used during the case interview with the parents or caregivers. Numerical and categorical variables were entered into Excel spreadsheets and analysed descriptively. R version 4.2.1 (R Core Team, Vienna, Austria) was used for all statistical analyses.

## Results

### Analysis of notification data

#### Age and sex

**Fig. 1:**
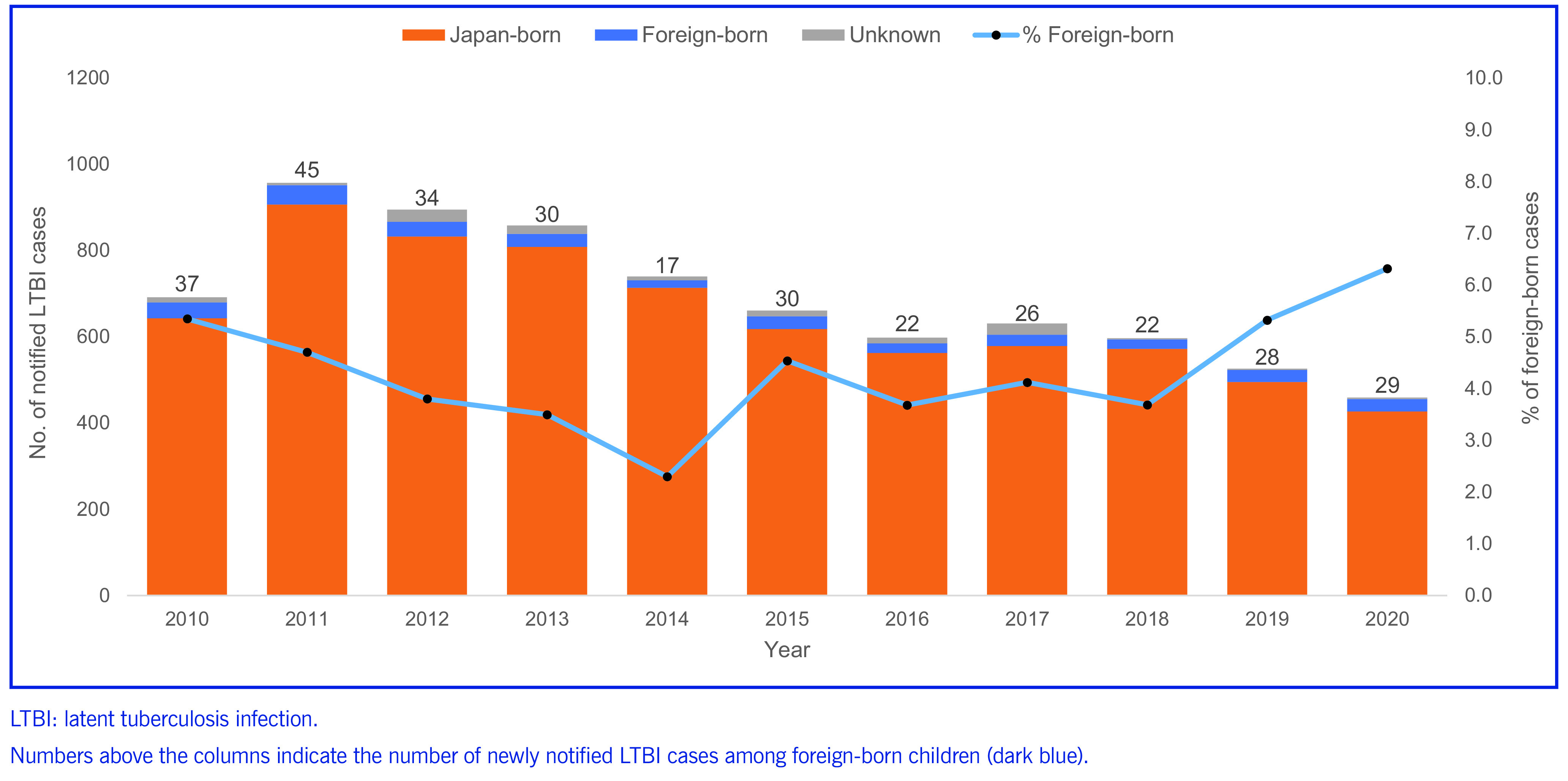
Number of new notifications of latent tuberculosis infection in children, by status as Japan-born or foreign-born and year, Japan, 2010–2020

There were 7160 Japan-born and 320 foreign-born children notified with LTBI in Japan between 2010 and 2020. During this time, the annual number of case notifications in Japan declined, while the proportion of foreign-born children among all notified cases in children declined until 2014 and then increased (**Fig. 1**). In 2020, 29 cases of LTBI were notified among foreign-born children, which was 6.3% of all LTBI cases in children.

[**insert** Figure 1]

**Fig. 2:**
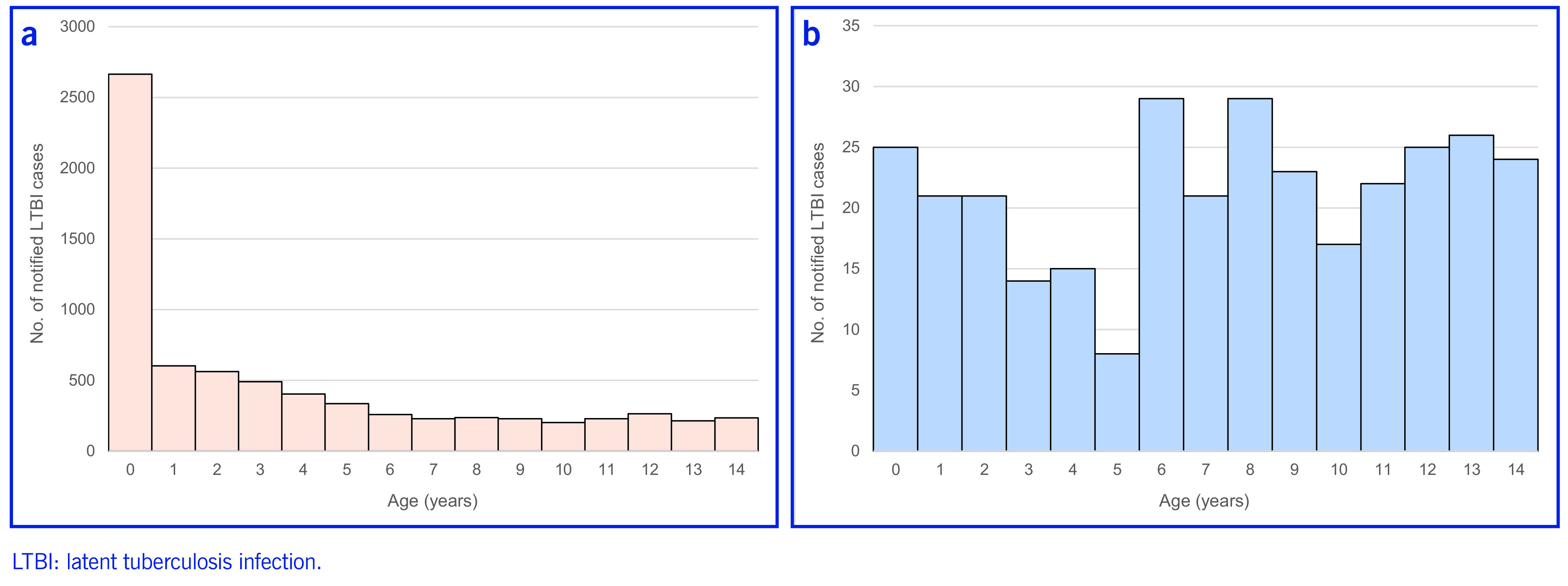
Age distribution of children notified with latent tuberculosis infection among (a) Japan-born children (n = 7160) and (b) foreign-born children (n = 320), Japan, 2010–2020

For Japan-born children, 37.1% (2663/7160) of notifications were among those aged < 1 year, with the number per year declining with age (data not shown). For foreign-born children, 70% (224/320) of the notifications were for children aged 5–14 years (**Fig. 2**). The average age of foreign-born children notified with LTBI was 7.3 years (standard deviation [SD]: ± 4.4 years), while for Japan-born children it was 3.8 years (SD: ± 4.4 years) (data not shown).

[**insert** Figure 2]

#### Country of birth and year of entry to Japan for foreign-born children

The distribution of foreign-born children notified with LTBI by country of birth was 44.1% (141/320) from the Philippines, 12.2% (*n* = 39) from China and 6.0% (*n* = 19) from Viet Nam. The year of entry into Japan was recorded for 157 of the 320 foreign-born children notified with LTBI, and of these children, 25.5% (*n* = 40) were diagnosed in the same year as their arrival, 26.8% (*n* = 42) were diagnosed 1 year after arrival, 28.0% (*n* = 44) were diagnosed 2–4 years after arrival and 19.8% (*n* = 31) were diagnosed 5 years after arrival (data not shown).

#### Mode of detection and source of infection

The difference in distribution by mode of detection was statistically significant between Japan-born and foreign-born children notified with LTBI (**Table 1**; *P* < 0.001). For both Japan-born and foreign-born children, the majority of LTBI cases notified were contacts of patients with active TB in the same household. A higher proportion of foreign-born cases notified with LTBI were detected through routine school health check-ups compared with Japan-born case notifications (20.3% vs 0.5%, *P* < 0.001), and there were higher proportions of Japan-born cases diagnosed during other contact investigations and in clinical settings compared with foreign-born cases notified (19.4% vs 10.9%, *P* = 0.002 for other contact investigations; 14.0% vs 8.1%, *P* = 0.004 for clinical settings) (**Table 1**).

**Table 1:** Mode of detection and possible source of infection for notifications of latent tuberculosis infection in Japan-born and foreign-born children, Japan, 2010–2020

Characteristic	No. (%) of children				*P*
	Japan-born		Foreign-born		
**Total**	7160	(100.0)	320	(100.0)	-
**Mode of detection**	-	-	-	-	< 0.001
Household contact investigation	3718	(51.9)	156	(48.8)	-
Other contact investigation	1390	(19.4)	35	(10.9)	-
School health check-up	33	(0.5)	65	(20.3)	-
Other mass health check-up	125	(1.7)	4	(1.3)	-
Clinical setting	1001	(14.0)	26	(8.1)	-
Other or unknown	893	(12.5)	34	(10.6)	-
**Source of infection**	-	-	-	-	< 0.001
Mother	671	(9.4)	47	(14.7)	-
Father	516	(7.2)	18	(5.6)	-
Grandparent	875	(12.2)	17	(5.3)	-
Sibling	32	(0.4)	11	(3.4)	-
School	212	(3.0)	3	(0.9)	-
Friends (outside school)	34	(0.5)	1	(0.3)	-
Hospital	81	(1.1)	0	(0.0)	-
Other	488	(6.8)	12	(3.8)	-
Unknown	4251	(59.4)	211	(65.9)	-

The reported source of infection was available for 40.6% (2909/7160) of Japan-born and 34.1% (109/320) of foreign-born LTBI notifications in children, and the difference in distribution by source of infection between the two groups was statistically significant (**Table 1**; *P* < 0.001). The proportion of notifications with grandparents as the source of infection was higher for Japan-born patients (12.2% vs 5.3%, *P* = 0.002), while the proportions of notifications with mothers or siblings as the source of infection were higher for foreign-born patients (14.7% vs 9.4%, *P* < 0.001 for mothers; 3.4% vs 0.4%, *P* < 0.001 for siblings) (**Table 1**).

### Treatment outcomes

Data on treatment outcomes between 2016 and 2019 were available for 2187 Japan-born and 99 foreign-born cases. Of these, 2162 Japan-born and 98 foreign-born cases had started LTBI treatment. The difference in treatment outcomes between the Japan-born and foreign-born cases was not statistically significant (*P* = 0.979), with 91.6% (1980/2162) of Japan-born and 89.8% (88/98) of foreign-born cases completing their treatment (**Table 2**).

**Table 2:** Treatment outcomes for notifications of latent tuberculosis infection in Japan-born and foreign-born children who had started treatment, Japan, 2016–2019

Treatment outcome	No. (%) of children		*P*
	Japan-born	Foreign-born	
Total^a^	2162 (100.0)	98 (100.0)	0.979
Completed	1980 (91.6)	88 (89.8)	-
Died	1 (0.0)	0 (0.0)	-
Failed	3 (0.1)	0 (0.0)	-
Lost to follow-up	58 (2.7)	3 (3.1)	-
Transferred out	47 (2.2)	3 (3.1)	-
Still on treatment	67 (3.1)	4 (4.1)	-
Unknown	6 (0.3)	0 (0.0)	-

^a^ A total of 25 Japan-born children and one foreign-born child had not started treatment at the time of analysis; they are not included in the analysis of treatment outcomes.

### Survey of public health centre staff

In 2019, 27 foreign-born children were notified with LTBI from 21 PHCs. A questionnaire survey was sent to these PHCs, of which 16 responded about 23 children. For all notifications, face-to-face case interviews were conducted upon registration by public health nurses with parents or caregivers; none of the interviews were conducted with the children themselves.

**Table 3** summarizes the nationalities of parents or caregivers (as a foreign national or Japanese national) and the language used for the interview. Among the 10 children who had one foreign-born parent, the interview was conducted with the Japanese parent for four cases, with the non-Japanese parent for four cases and with Japanese-speaking relatives for two cases. Interviews with foreign-born parents were conducted in Japanese without an interpretation service for three cases and in Tagalog for one case (**Table 3**).

**Table 3:** Language spoken during case interview for notifications of latent tuberculosis infection in foreign-born children, by nationality of their parents, Japan, 2019

One parent is a foreign national	10	9	1	0
Both parents are foreign nationals^a^	12	7	3	1
Both parents are Japanese nationals	1	1	0	0
Total	23	17	6	1

^a^ No information was provided about the language used during the interview for two of the cases.

Among the 12 children whose parents were both foreign nationals, the interview was conducted in Japanese for seven cases. No interpretation assistance was provided, except for one case in which the public health nurse used a mobile translation application. An informational leaflet was used during the interview for one case, and the leaflet was in Japanese (**Table 3**). For three children, the interview was conducted in the parents’ native language with the assistance of a friend or acquaintance, none of whom were professional medical interpreters. No translation apps or other tools were used. For the remaining two children, the language of the interview was not reported.

The final case had two Japanese parents and their interview was conducted in Japanese.

## Discussion

Our study is the first to explore the characteristics of foreign-born children notified with LTBI in Japan. Compared with Japan-born children, foreign-born children notified as having LTBI were more likely to be older, have their mother or sibling as their source of infection and have LTBI detected via a routine school health check. That the source of infection was a first-degree relative may be due to visa regulations, as foreign-born persons working in Japan are often permitted to bring only their spouse and child (or children) and, therefore, usually live in a nuclear family. The detection of LTBI in foreign-born children during school health checks is likely due to health workers following the manual on TB prevention in schools, (*5*) which recommends tuberculin skin testing (TST) or interferon-γ release assay (IGRA) testing for children from countries with a high TB burden upon entry to primary school and LTBI treatment for those who test positive.

In the majority of countries where these children were born (i.e. countries with a high TB burden), routine LTBI screening is not conducted. Rather, LTBI treatment is usually offered only to children aged ≤ 5 years who are household contacts of active TB cases, after active TB has been ruled out, but neither TST nor IGRA are usually conducted as part of household contact investigations. (*6*-*8*) Therefore, it is expected that many foreign-born children and their parents in Japan are unfamiliar with the experience of being tested for and diagnosed with LTBI, and even less familiar with this in school settings. Thus, PHCs are required to provide education to patients and families and care that accounts for these differences.

Furthermore, previous studies have shown that children may face different barriers to initiating and completing LTBI care compared with adults. (*9*-*11*) Some barriers are patient-related factors, such as knowledge, concerns about side-effects and the school environment, which may be important to older children. (*12*, *13*) However, especially with younger children, treatment decisions are made by parents or caregivers, and their knowledge and perceptions regarding TB infection, (*9*) the adverse effects of medication (*14*, *15*) and medical contraindications to treatment, (*16*, *17*) personal health beliefs (*13*, *18*, *19*) and relationship with their children (*20*) have been shown to play important roles in treatment completion. In studies from lower-income countries, socioeconomic factors have also been identified as barriers to treatment completion, such as low monthly income, (*9*) high cost of transport (*9*) and conflicts with work schedules, (*13*) which all place burdens on parents or caregivers.

Our results showed that the case interviews at PHCs were largely conducted in Japanese, with limited use of language interpretation services, either in person or via an app, even when neither of the parents were Japanese nationals. Previous studies have repeatedly shown there is limited availability of medical interpretation services for foreign-born patients with (*21*, *22*) and without TB (*23*, *24*) in Japan and that language is a major barrier to accessing health care for foreign-born persons in Japan.

Our study is not without limitations, and the most significant is that the study was limited to children notified with LTBI to the JTBS system. Therefore, it was unable to capture those children who, although eligible for LTBI treatment, were not notified and thus had not started treatment. The JTBS system also did not capture the total number of children tested for LTBI nor those who tested negative. A scoping review on care cascades for paediatric LTBI has identified several stages during which dropout from the cascade could occur. (*24*) In Japan, too, it is quite possible that some foreign-born children who are eligible never begin LTBI treatment. A comprehensive study to capture the care cascade for foreign-born children diagnosed with LTBI, both before travelling to and after entering Japan, is needed. Second, our survey specifically focused on the language spoken during the case interview and did not explore other issues, such as knowledge and attitudes towards and practices around LTBI between Japan-born and foreign-born children and their parents. Further studies are needed to explore these differences.

Our study indicated that foreign-born children notified with LTBI tended to be older, have their mother or sibling identified as the source of infection, and be detected via a routine school health check. The use of language interpretation services by health-care providers and the parents or caregivers of children diagnosed with LTBI in 2019 was limited. This may lead to poorer communication, knowledge and understanding about TB infection and the necessity for preventive treatment, as well as a lack of trust. Action is needed to address long-standing issues around language barriers for foreign-born persons, both children and adults, in Japan.

## References

[1] Tuberculosis Surveillance Center. Tuberculosis in Japan: annual report 2021. Tokyo: Department of Epidemiology and Clinical Research, the Research Institute of Tuberculosis; 2021. Available from: https://jata-ekigaku.jp/english/tb-in-japan, accessed 4 October 2022.

[2] Kawatsu L, Uchimura K, Ohkado A. Trend and treatment outcomes of latent tuberculosis infection among migrant persons in Japan: retrospective analysis of Japan tuberculosis surveillance data. BMC Infect Dis. 2021 Jan 9;21(1):42. 10.1186/s12879-020-05712-1 33422003 PMC7796533

[3] Nishimura T, Ota M, Mori M, Hasegawa N, Kato S, Kawanabe H. Tuberculosis infection survey of international students. Bull Keio Univ Health Cent. 2017;35(1):37–40. [in Japanese]

[4] Nishimura T, Mori M. Tuberculosis infection survey of international students: the second report. Bull Keio Univ Health Cent. 2019;37(1):35–9. [in Japanese]

[5] Manual on tuberculosis prevention in schools Tokyo: Ministry of Education, Culture, Sports, Science and Technology; 2012 (in Japanese). Available from: https://www.mext.go.jp/component/a_menu/education/detail/__icsFiles/afieldfile/2012/03/30/1318847_04.pdf, accessed 10 August 2022.

[6] National Tuberculosis Control Program manual of procedures, sixth edition. Manila: National Tuberculosis Control Program, Department of Health, Philippines; 2020. Available from: https://doh.gov.ph/node/5111, accessed 15 March 2023.

[7] National tuberculosis management guidelines 2019. Thimi (Nepal): Department of Health Services, Ministry of Health and Population, Government of Nepal; 2019. Available from: https://nepalntp.gov.np/wp-content/uploads/2019/10/National-Tuberculosis-Management-Guidelines-2019_Nepal.pdf, accessed 15 March 2023.

[8] Technical guidelines for handling latent tuberculosis infection in 2020. Jakarta: Ministry of Health, Republic of Indonesia; 2020 (in Indonesian). Available from: https://tbindonesia.or.id/pustaka_tbc/petunjuk-teknis-penanganan-infeksi-laten-tuberkulosis-tahun-2020/, accessed 15 March 2023.

[9] Séraphin MN, Hsu H, Chapman HJ, de Andrade Bezerra JL, Johnston L, Yang Y, et al. Timing of treatment interruption among latently infected tuberculosis cases treated with a nine-month course of daily isoniazid: findings from a time to event analysis. BMC Public Health. 2019 Sep 3;19(1):1214. 10.1186/s12889-019-7524-4 31481046 PMC6724263

[10] Saunders MJ, Koh GC, Small AD, Dedicoat M. Predictors of contact tracing completion and outcomes in tuberculosis: a 21-year retrospective cohort study. Int J Tuberc Lung Dis. 2014 Jun;18(6):640–6. 10.5588/ijtld.13.0486 24903932

[11] Hovell M, Blumberg E, Gil-Trejo L, Vera A, Kelley N, Sipan C, et al. Predictors of adherence to treatment for latent tuberculosis infection in high-risk Latino adolescents: a behavioral epidemiological analysis. Soc Sci Med. 2003 Apr;56(8):1789–96. 10.1016/S0277-9536(02)00176-4 12639595

[12] Hill L, Blumberg E, Sipan C, Schmitz K, West J, Kelley N, et al. Multi-level barriers to LTBI treatment: a research note. J Immigr Minor Health. 2010 Aug;12(4):544–50. 10.1007/s10903-008-9216-5 19085104 PMC2904450

[13] Silva AP, Hill P, Belo MT, Rabelo SG, Menzies D, Trajman A. Non-completion of latent tuberculous infection treatment among children in Rio de Janeiro State, Brazil. Int J Tuberc Lung Dis. 2016 Apr;20(4):479–86. 10.5588/ijtld.15.0609 26970157

[14] Villarino ME, Scott NA, Weis SE, Weiner M, Conde MB, Jones B, et al.; International Maternal Pediatric and Adolescents AIDS Clinical Trials Group; Tuberculosis Trials Consortium. Treatment for preventing tuberculosis in children and adolescents: a randomized clinical trial of a 3-month, 12-dose regimen of a combination of rifapentine and isoniazid. JAMA Pediatr. 2015 Mar;169(3):247–55. 10.1001/jamapediatrics.2014.3158 25580725 PMC6624831

[15] Chang SH, Eitzman SR, Nahid P, Finelli ML. Factors associated with failure to complete isoniazid therapy for latent tuberculosis infection in children and adolescents. J Infect Public Health. 2014 Mar-Apr;7(2):145–52. 10.1016/j.jiph.2013.11.001 24361084

[16] Diallo T, Adjobimey M, Ruslami R, Trajman A, Sow O, Obeng Baah J, et al. Safety and side effects of rifampin versus isoniazid in children. N Engl J Med. 2018 Aug 2;379(5):454–63. 10.1056/NEJMoa1714284 30067928

[17] Huang H, Yuan G, Du Y, Cai X, Liu J, Hu C, et al. Effects of preventive therapy for latent tuberculosis infection and factors associated with treatment abandonment: a cross-sectional study. J Thorac Dis. 2018 Jul;10(7):4377–86. 10.21037/jtd.2018.06.138 30174886 PMC6105962

[18] Li Y, Zhou C, Zheng Y, Hong J, Yang M, Yuan Z, et al. The acceptability and feasibility of chemical prophylaxis for schoolchildren and adolescents with latent tuberculosis infection in Shanghai, China: a qualitative study. Int J Infect Dis. 2016;45 Suppl. 1:397. 10.1016/j.ijid.2016.02.849

[19] Hamdi B, Blibech H, Berraies A, Mazaoui S, Ammar J, Hamzaoui A. Evaluation of adherence to tuberculosis screening in children. Eur Respir J. 2016;48:PA1284. 10.1183/13993003.congress-2016.PA1284

[20] Takayanagi K, Nagata Y, Ota M. Tuberculosis among foreign-born technical intern trainees in Japan: a questionnaire survey, 2018. Kekkaku. 2022;97:155–61. [in Japanese]

[21] Ohkado A, Kawatsu L, Hamaguchi Y, Yamaguchi A, Uchimura K. [A survey of foreign-born tuberculosis or latent tuberculosis infection patients who transferred-out of Japan during treatment: (2) cross-border referral]. Kekkaku. 2022;97:33–9. [in Japanese]

[22] Tanaka Y, Kato K, Uerara J, Uto M, Nakamura Y. [Current status and issues in dealing with foreigners at insurance-participating medical facilities: a prefectural survey of hospitals and municipal health centers in Mie Prefecture]. J Jpn Soc Travel Health. 2017;11(2):74–80. [in Japanese]

[23] Hamai T, Nagata A, Nishikawa H. [The need for medical interpreters: a questionnaire survey of municipal hospitals in Japan]. Nihon Koshu Eisei Zasshi. 2017;64(11):672–83 (in Japanese). pmid:29249778. 10.11236/jph.64.11_672 29249778

[24] Campbell JI, Sandora TJ, Haberer JE. A scoping review of paediatric latent tuberculosis infection care cascades: initial steps are lacking. BMJ Glob Health. 2021 May;6(5):e004836. 10.1136/bmjgh-2020-004836 34016576 PMC8141435

